# USING GENERATIVE AI TO ENHANCE BEHAVIORAL INTERVENTIONS FOR CARE PARTNERS OF OLDER ADULTS

**DOI:** 10.1093/geroni/igae098.1919

**Published:** 2024-12-31

**Authors:** Karla Washington, George Demiris, Jina Huh-Yoo, Rezvaneh Rezapour, Lu Wang, Elham Aghakhani, Max Song

**Affiliations:** Washington University in St. Louis, St. Louis, Missouri, United States; University of Pennsylvania, Philadelphia, Pennsylvania, United States; Drexel University, Philadelphia, Pennsylvania, United States; Drexel University, Philadelphia, Pennsylvania, United States; Drexel University, Philadelphia, Pennsylvania, United States; Drexel University, Philadelphia, Pennsylvania, United States; Drexel University, Philadelphia, Pennsylvania, United States

## Abstract

Care partners play an essential role in providing long-term care to community-dwelling older adults, offering vital support to individuals who require or desire assistance with instrumental and other activities of daily living. However, care partners’ responsibilities often entail significant physical, emotional, and financial strain, resulting in compromised health and well-being. Numerous studies support the efficacy of behavioral interventions in reducing the deleterious health outcomes often associated with serving as a care partner, but the resource-intensive nature of many such interventions introduces numerous implementation barriers. This presentation will explore the promise and potential pitfalls of using generative AI to enhance the delivery of behavioral interventions for care partners of older adults. Presenters will supplement a review of relevant literature with specific examples drawn from their own research using a large language model (ChatGPT 3.5) to enhance the delivery of PISCES (Problem-solving Intervention to Support Caregivers in Everyday Situations), a cognitive-behavioral intervention developed for care partners of older adults with serious illness. Generative AI’s potential as a tool to deliver the intervention’s psychoeducational content and also to monitor intervention fidelity demonstrated by human interventionists will be described, as will researchers’ intentional efforts to ensure the tool does not perpetuate problematic (e.g., ageist) perspectives based on an analysis of 250 transcribed intervention sessions used to train it. The presentation will conclude with actionable practice recommendations that harness the potential of this rapidly emerging technology in an ethically sound manner that enhances the equitable delivery of evidence-based care partner support for all.

